# The effect of pneumoperitoneum on the cross-sectional areas of internal jugular vein and subclavian vein in laparoscopic cholecystectomy operation

**DOI:** 10.1186/s12871-016-0226-x

**Published:** 2016-08-11

**Authors:** Hüseyin Ulaş Pınar, Rafi Doğan, Ümmü Mine Konuk, Egemen Çifci, Enes Duman, Erdal Karagülle, Emin Türk, Ömer Karaca

**Affiliations:** 1 Baskent University Konya Research and Training Center Anesthesiology Department, Hocacihan mah. Saray cad. no:1, Selçuklu, Konya 42080 Turkey; 2 Baskent University Konya Research and Training Center Radiology Department, Hocacihan mah. Saray cad. no:1, Selçuklu, Konya 42080 Turkey; 3 Baskent University Konya Research and Training Center General Surgery Department, Hocacihan mah. Saray cad. no:1, Selçuklu, Konya 42080 Turkey

## Abstract

**Background:**

Increased central venous pressure secondary to an increase in intraabdominal pressure has been reported during laparoscopic surgery. However, no study has yet determined the effect of pneumoperitoneum on cross-sectional area (CSA) of central veins by ultrasonography during laparoscopic cholecystectomy. Herein, we aimed to quantify changes in CSAs of internal jugular (IJV) and subclavian veins (SCV) by ultrasonography during this surgery.

**Methods:**

This study included 60 ASA I-II patients scheduled for laparoscopic cholecystectomy surgery under general anesthesia. Pneumoperitoneum was performed with CO_2_ at 12 mmHg. The CSAs of right IJV and right SCV were measured using a 6 Mhz ultrasonography transducer in supine and neutral positions before anesthesia induction (T1), 5 min after connecting to mechanical ventilator (T2), 5 min after creation of pneumoperitoneum (T3), at the end of pneumoperitoneum (T4), and 5 min after desufflation and before extubation (T5) both at end-expiration and end-inspiration.

**Results:**

The comparison of IJV CSA at inspiration showed significant increase in T3 value compared to T2 value (*p* < 0.001). Similarly the expiratory measurements of IJV CSA demonstrated significant increase in T3 value compared to T2 value (*p* < 0.001). The comparison of inspiratory CSA measurements of SCV showed significantly increased in T3 (*p* = 0.009) than T2 value. In expiratory measurements there was a significant increase in T3 (*p* = 0.032) value compared to T2. All measurements of IJV and SCV SCAs both end-inspiration and end-expiration T5 values significantly decreased compared to T4 values (*p* < 0.001).

**Conclusions:**

Pneumoperitoneum with an intraabdominal pressure of 12 mmHg produces significant increases in IJV and SCV CSAs during laparoscopic cholecystectomy procedure. We believe that this finding may enhance our understanding of pneumoperitoneum-induced hemodynamic changes and facilitate catheterization attempts.

**Trial registration:**

Date of registration 21/07/2016, ISRCTN Registry ( No:ISRCTN15164056, registered retrospectively).

## Background

Central venous catheter placement is a widely practiced medical procedure [1]. Central venous catheters are commonly used by the intensive care and anesthesiology disciplines for the purpose of hemodynamic monitorization, drug administration, and parenteral nutrition especially for hemodynamically unstable patients or those who are scheduled to undergo major surgery [2]. Internal jugular vein (IJV) and subclavian vein (SCV) are the most commonly used veins for central venous catheterization. It is of importance to increase the cross-sectional area (CSA) of the target vein to facilitate central venous catheter insertion. To date, many studies have explored factors affecting CSA of main venous structures. It has been reported that positive end-expiratory pressure (PEEP) application [1, 3], intrathoracic pressure application [4], Trendelenburg position [1, 4], hepatic compression [4], and valsalva maneuver [5] increase the CSA of IJV. The CSA of SCV, on the other hand, is reportedly increased by the combined use of inspiratory hold and Trendelenburg position [6], complete expiration in spontaneously breathing subjects [7], Trendelenburg position alone [8], and leg elevation [9].

During laparoscopic cholecystectomy operation, CSAs of IJV and SCV may be expected to increase owing to increased intraabdominal pressure by CO_2_ insufflation. Although central venous pressure has been reported to increase [10, 11], no study to date has ever performed CSA measurements during pneumoperitoneum under laparoscopic cholecystectomy. This study aimed to investigate the presence and magnitude of IJV or SCV CSAs change elicited by pneumoperitoneum resulting from CO_2_ insufflation during laparoscopic cholecystectomy operation.

## Methods

This study was approved by Baskent University, Institutional Review Board and Ethics Committee (Project No: 13/254 and Chairperson: Prof. Dr. Hakan Özkardeş) and supported by Baskent University Research Fund. All patients gave an informed consent before study participation. The study was conducted at a tertiary university hospital between February 2015 and September 2015. It included a total of 60 ASA (American Society of Anesthesiology) group I-II patients aged 25–70 years who were scheduled to undergo cholecystectomy surgery with laparoscopic method.

**Fig. 1:**
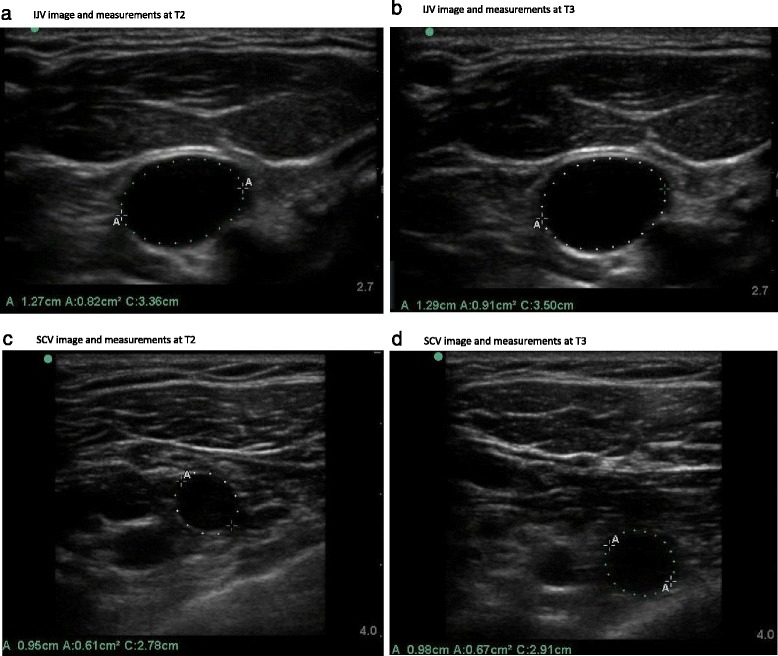
Images from a patient’s IJV and SCV measurements by time point T2 and T3. **a** IJV image and measurements at T2. **b** IJV image and measurements at T3. **c** SCV image and measurements at T2. **d** SCV image and measurements at T3

Patients with cardiovascular disease, severe COPD, chest wall deformity, and a history of use of any drug altering vascular tonus, or a history of neck, clavicle, lung, great vessel, or chest wall surgery were excluded. Age, height, weight, and body mass index of each patient was recorded. The patients fasted for 8 h before the operation and were hydrated with isotonic saline infusion at a rate of 2 ml/kg/h. All patients were administered oral alprazolam 0.5 mg the night before the operation. All patients were administered general anesthesia. Anesthesia induction was achieved by i.v. propofol 2 mg/kg, i.v. fentanyl 1 μgr/kg, and i.v. rocuronium 0.6 mg/kg, and endotracheal intubation was followed anesthesia induction. Anesthesia maintenance was achieved by sevoflurane 1–2 % and i.v. remifentanyl 0.1–0.5 μgr/kg/min infusion. Mechanical ventilation was provided in the volume-controlled mode with an airway pressure not exceeding 20 cmH_2_O, providing a tidal volume of 6–7 ml/kg, respiratory rate of 12/min, and %40/%60 O_2_/air mixture. ECG, peripheral oxygen saturation, and noninvasive blood pressure monitorization were performed during surgery. All patients were applied pneumoperitoneum in supine position via CO_2_ insufflation (flow rate 2–4 L/min) to achieve an intraabdominal pressure of 12 mmHg. Respiratory rate was adjusted to reach an end-tidal carbon dioxide level of 30–35 mmHg during surgery. CSAs and diameters of right SCV and right IJV were measured in supine position on operating table at both end-expiration and end-inspiration by an ultrasonography device (M-TurboTM; Fujifilm SonoSite Inc., Washington, United States) using a 6 MHz two-dimensional flat ultrasonography transducer (band width of 13–6 MHz, depth 6 cm) before anesthesia induction (Control, T1), 5 min after intubation and connection to mechanical ventilator (T2), 5 min after creating pneumoperitoneum (T3), at the end of pneumoperitoneum (T4), and after desufflation and before extubation (T5). Figure 1 shows a patient’s measurements and images of both IJV and SCV.

IJV was evaluated by placing the ultrasonography transducer longitudinally on skin at the level of cricoid cartilage without applying pressure. SCV diameter was measured by placing the same transducer at the junction of the medial and middle third of the clavicle, corresponding to the percutaneous needle entry area for SCV catheterization. The venous measurements included vein diameter, circumference, and cross-sectional area. All measurements were performed while the patients were in supine and neutral positions. The amount of intraoperatively administered fluids and duration of operation were recorded.

### Statistical analysis

As previous studies with a power analysis performed considered a 20 % increase in IJV CSA significant, we planned to enroll at least 58 patients to obtain a confidence level of 95 %, and we ultimately enrolled a total of 60 patients. Study data were analyzed using SPSS (Statistical Package for Social Science) for Windows 11.5 software package. Kolmogorov Smirnov test was used to test the normality of continuous numeric variables. Descriptive statistics included mean ± standard deviation for continuous numeric variables, and number and percentage (%) for nominal variables. Only, T2–T3 and T4–T5 measurements were compared with each other. Normally distributed measurements were compared by two-paired samples t-test and non-normal distributed measurements were compared by nonparametric two-related samples Wilcoxon test. The results were considered statistically significant when *p* < 0.05.

## Results

**Fig. 2:**
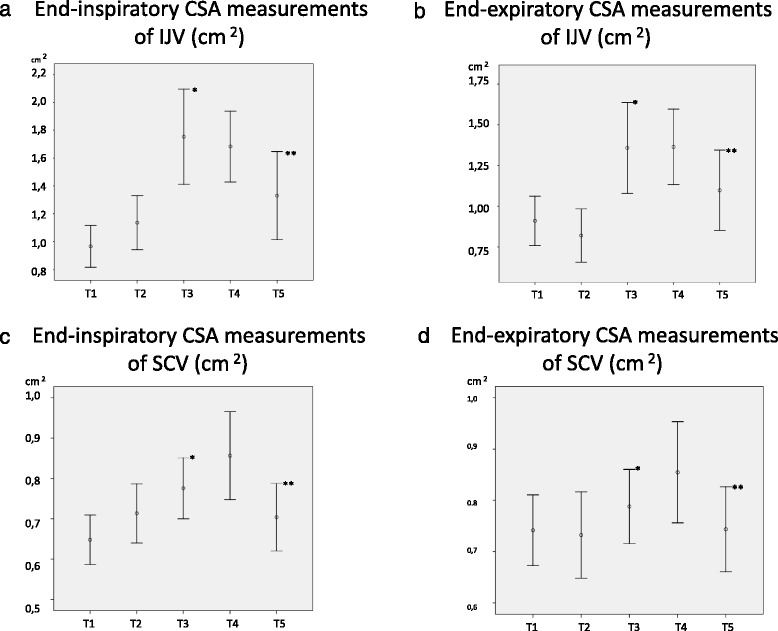
End-inspiratory and endexpiratory measurements of CSAs of IJV, SCV. **a** End-inspiratory CSA measurements of IJV (cm^2^) T1. **b** End-expiratory CSA measurements of IJV (cm^2^). **c** End-inspiratory CSA measurements of SCV (cm^2^). **d** End-expiratory CSA measurements of SCV (cm^2^). * Statistical significant increase when compared to T2 value. ** Statistical significant decrease when compared to T4 value

Sixty patients were included in the study. The demographic properties of the study population were shown on Table 1. The same table also specifies intraoperatively administered fluid volumes and operative times. The statistical comparisons of the inspiratory CSA measurements of IJV showed that there was significant increase (54 % increase) in T3 compared to T2 value (*p* < 0.001). The expiratory measurements of IJV CSAs demonstrated significant increase in T3 value compared to T2 value (*p* < 0.001) (64 % increase). The comparison of inspiratory CSA measurements of SCV showed significantly increased T3 value than T2 value (*p* = 0.009). In expiratory measurements on the other hand, there was a significant increase in T3 ( *p* = 0.032) compared to T2 (Table 2). All measurements of IJV and SCV SCAs both end-inspiration and end-expiration T5 values significantly decreased compared to T4 values (*p* < 0.001). These results were graphically depicted in Fig. 2.

**Table 1:** Demographic and clinical properties of the study population

Variables	*n* = 60
Age (years)	44.4 ± 15.6
Age range (years)	19–70
Sex	
Male	25 (%41.7)
Female	35 (%58.3)
Height (cm)	166.8 ± 9.8
Weight (kg)	80.6 ± 15.3
Body mass index (kg/m^2^)	29.1 ± 5.2
ASA	
I	34 (%56.7)
II	26 (%43.3)
Operation time (min)	53.5 ± 14.8
Amount of intraoperative fluid replacement (ml)	238.2 ± 41.9

The values were presented as mean ± standard deviation

**Table 2:** Comparisons of T2–T3 and T4–T5 periods of cross-sectional area measurements

	T2	T3	T4	T5
IJV-I CSA (cm^2^)	1,13 ± 0,738	1,75 ± 1,296 (*t* = −5,886, *p* < 0,001)^b^	1,68_±_0,966	1,33 ± 1,204^a^ (*Z* = −5,162, *p* < 0,001)^c^
IJV-E CSA (cm^2^)	0,82 ± 0,622	1,35 ± 1,061 (*t* = −5,667, *p* < 0,001)^b^	1,36 ± 0,877	1,09 ± 0,933^a^ (*Z* = −4,205, *p* < 0,001)^c^
SCV-I CSA (cm^2^)	0,71 ± 0,276	0,77_±_0,286 (*t* = −2,704, *p* = 0,009)^b^	0,85 ± 0,410^a^	0,70_±_0,315 (*Z* = −3,965, *p* < 0,001)^c^
SCV-E CSA (cm^2^)	0,73 ± 0,317	0,78 ± 0,273 (*t* = −2,205, *p* = 0,032)^b^	0,85 ± 0,371	0,74 ± 0,312 (*t* = 3,864, *p* < 0,001)^c^

The values were presented as mean ± standard deviation

*IJV* Internal juguler vein, *SCV* subclavian vein, *I* Inspiration, *E* Expiration, *CSA* Cross-sectional area

^a^ It is not normally distributed

^b^ Statistical significance for comparison of T3 and T2 value

^c^ Statistical significance for comparison of T5 and T4 value

## Discussion

This trial studied the effects of pneumoperitoneum on the CSA measurements of IJV and SCV. According to the results of the study, pneumoperitoneum applied with a pressure of 12 mmHg caused significant increases in CSA of IJV and SCV at both expiration and inspiration. After desufflation of intraabdominal gas IJV and SCV CSAs decreased significantly. Measurements at desufflation period showed greater IJV CSA than basal measurements and this probably resulted from an elevated intrathoracic pressure due to mechanical ventilation.

The effect of manuvers increasing intraabdominal pressure has been previously studied. Hepatic compression [4] and increased valsalva maneuver [5] have been similarly reported to increase CSA. The hemodynamic effects of pneumoperitoneum, on the other hand, are somewhat more complicated. Increased intraabdominal pressure can be considered to have a biphasic effect on venous return [11]. It is hypothesized that an increase of venous return occurs as a result of the compression of abdominal capacitance veins initially, followed by decrease in it as a result of increased resistance against venous return in the abdomen and extremities. However, some studies have suggested that when right atrial pressure is normal to high, that is when the patient is not hypovolemic, pneumoperitoneum increases venous return; it may be reasonable to argue that such studies have conveyed a more accurate information [12]. We felt no necessity to discuss the hemodynamic effects of severe hypovolemia and cardiovascular disorders as none of our patients had these morbidities.

Pneumoperitoneum affects cardiovascular system by two ways, namely by direct pressure effect and hypercarbia. However, alterations induced by hypercarbia are less pronounced compared to those induced by the mechanical effects of increased intraabdominal pressure [12]. We prevented hypercarbia by adjusting end-tidal CO_2_ controlled respiratory rate. Hemodynamic changes secondary to increased intraabdominal pressure by gas insufflation vary by several factors including intravascular volume, the magnitude of intraabdominal pressure, and patient position. In this study, we tried to standardize these variables by making measurements in supine position, administering i.v. fluids during the fasting period, performing a standard intraabdominal pressure application, and adjusting respiratory rate by EtCO_2_ control.

Since no different pneumoperitoneum pressures were used in our study, their effect could not be assessed. However, prior studies have shown that different pressures have variable hemodynamic effects [13]. Dexter et al. randomized subjects undergoing laparoscopic cholecystectomy into two groups with pneumoperitoneum pressures of 7 mmHg and 15 mmHg. They found heart rate and mean arterial pressure increase in both groups, but stroke volume and cardiac output were significantly reduced in the 15 mmHg group (10 and 26 %, respectively) [14]. McLaughlin et al. reported a 30 % reduction in cardiac output and stroke volume and a 60 % increase in mean arterial pressure after the application of a 15-mmHg pneumoperitoneum compared to the pre-insufflation period [15]. Another study showed that pressures below 15 mmHg increased venous return by squeezing venous bed while pressures of 15 mmHg or above caused a reduction in venous return and blood pressure as a result of inferior vena cava compression [16].

Moreover, the effect of intubation and mechanical ventilation on intrathoracic pressure should not be overlooked. That is, combined effect of increased intraabdominal and intrathoracic pressures may have contributed to CSA increase. This may explain the lack of a biphasic central vein CSA change despite pneumoperitoneum’s biphasic effect on venous return.

Central catheters are not routinely used for monitorization of patients with a low ASA category in laparoscopic cholecystectomy operations. However, they may be necessary in case of severe cardiovascular insufficiency or respiratory complications. Although USG is now widely utilized for central catheterization, the latter is performed blindly by using reference points at many centers. A knowledge of diameter change in central veins will reduce the rate of complications in laparoscopic surgeries. According to the results of our study, central venous intervention can be more easily performed after laparoscopic insufflation in such patients. Future studies may investigate if CSA increments will be more exaggerated when maneuvers producing CSA increments, such as PEEP or Trendelenburg position, are applied in conjunction with laparoscopy, or whether different CSA increments will be obtained when different intraabdominal pressure limits are used.

This study had some limitations. First of all, as the patients were undergoing cholecystectomy operation, they were placed in head up and left lateral tilt position after CO_2_ insufflation and trocar’s placement at the first stage of laparoscopy procedure. Therefore, the study was so designed that the measurements could only be made at the first stage of laparoscopy and 5 min after placing patients again in supine position following the end of the procedure. A study that would be conducted on patients continuously remaining in neutral position may allow performing more frequent measurements that would facilitate the observation of possible changes in every stages of laparoscopy procedure. As a result, after we observed the effect of intraabdominal pressure increase on vein CSAs, we decided to investigate the correlation of CSA changes with pressure alterations in abdominal compartment syndrome in a future study.

## Conclusions

In conclusion, pneumoperitoneum applied with a pressure of 12 mmHg during laparoscopic cholecystectomy produced significant increases in the CSAs of IJV and SCV. More studies are needed to study the clinical effects of this result, such as those on catheter placement time and the incidence of side effects, in patients necessitating catheter placement.

## Abbreviations

ASA, American society of anesthesiologists; CSA, cross-sectional area; IJV, internal jugular vein; PEEP, positive end expiratory pressure; SCV, subclavian vein.
